# Attenuation without protective immunogenicity: effects of codon-pair deoptimization on foot-and-mouth disease virus serotype O in swine

**DOI:** 10.1038/s41598-026-61879-8

**Published:** 2026-07-16

**Authors:** Constantin Lorenz, Kira Wisnewski, Saskia Weber, Paul Deutschmann, Martin Beer, Michael Eschbaumer

**Affiliations:** Friedrich-Loeffler-Institut, Institute of Diagnostic Virology, 17493 Greifswald, Germany

**Keywords:** Foot-and-mouth disease, FMDV, Vaccine, Codon pair deoptimization, Animal challenge, Virus, Biotechnology, Diseases, Immunology, Microbiology

## Abstract

Foot-and-mouth-disease (FMD), caused by the FMD virus, is a contagious disease affecting cloven-hoofed animals. Since outbreaks often cause significant economic impact, control and prevention measures against FMDV, such as vaccines, are urgently needed especially in endemic regions. The use of inactivated whole-virus vaccines has led to the eradication of FMD in many industrialized nations. Nonetheless, recent outbreaks in Europe have highlighted the virus’s potential for rapid spread and its capacity to inflict substantial economic losses. Codon pair deoptimization is a novel strategy for viral attenuation. In this study, we report the first application of this approach to the P1 capsid coding region of FMDV serotype O. A recombinant virus carrying the deoptimized P1 region was attenuated in vitro, as evidenced by reduced replication capacity in porcine LFBK-αvβ6 cells. In vivo evaluations further demonstrated that a substantially higher dose was required to induce clinical disease in pigs than with wild-type viruses. However, immunization with lower and innocuous doses did not result in protective immunity. These findings underscore both the potential and the challenges of utilizing codon pair deoptimization for developing safe and effective FMDV vaccine candidates, emphasizing that further optimization is necessary to achieve a desirable balance between attenuation and immunogenicity.

**Supplementary Information:**

The online version contains supplementary material available at 10.1038/s41598-026-61879-8.

## Introduction

Foot-and-mouth disease (FMD) is a contagious viral disease that causes acute vesicular lesions in cloven-hoofed animals. The causative agent, the Foot-and-mouth disease virus (FMDV), is a member of the species *Aphthovirus vesiculae* within the *Picornaviridae* family. FMDV is endemic across vast regions of Africa and Asia. The virus has seven antigenically distinct serotypes, O, A, C, Asia 1, and SAT1–SAT3^1^. The positive-strand RNA virus genome of approximately 8,500 nucleotides has a single large open reading frame (ORF) flanked by untranslated regions. The ORF is divided into four main regions: the leader protein (Lpro) and the immature polyproteins P1, P2, and P3. Processing by three viral proteinases (Lpro, 2 A, 3Cpro) produces four structural proteins (1A/VP4, 1B/VP2, 1C/VP3, 1D/VP1) and ten non-structural proteins (Lpro, 2A, 2B, 2C, 3A, three copies of 3B/VPg, 3Cpro, and the RNA-dependent RNA polymerase 3Dpol).

An FMD outbreak in livestock can have severe socio-economic impacts, leading to significant financial losses due to trade restrictions, mass culling, and reduced production^2,3^. FMDV spreads through multiple transmission routes: infected domestic and wild animals can transmit the virus via direct contact or through contaminated animal products such as meat, milk, and semen. Additionally, the virus can be mechanically spread by personnel or equipment^4^. Recent FMDV outbreaks in Germany, Hungary and Slovakia (2025) underscore the continuing threat posed by the virus even in regions that had been free of the disease for a long time.

Vaccination against FMDV remains an important cornerstone in combating the disease. Current FMD control is predominantly achieved through chemically inactivated whole-virus vaccines^1^. While these vaccines are effective, they come with certain inherent disadvantages: the need to apply them by injection, a short period of protection of only a few months^5^, poor cross protection between different strains and serotypes, difficulty in differentiating infected from vaccinated animals (DIVA)^6^ and importantly, the high risk and expense associated with producing large amounts of infectious virus for inactivation in biocontainment facilities^7^.

Live attenuated vaccines (LAVs) represent a promising alternative due to the prospect of them inducing stronger and longer-lasting immune responses^8^. However, safety concerns, particularly the potential for regaining virulence, have historically limited their appeal, especially for already FMDV-free countries. Advances in synthetic biology have enabled novel strategies such as codon and codon-pair deoptimization^9^ which introduce numerous synonymous mutations into the viral genome without altering the resulting protein sequence. However, these mutations reduce translation efficiency and/or RNA stability, leading to viral attenuation. They have been successfully applied to attenuate viruses such as poliovirus and FMDV^10–12^.

This study explores the application of codon pair deoptimization to the P1 region of FMDV. While previous studies have focused on serotypes A and Asia 1^10,11^, we demonstrate this technique with serotype O, the most widespread FMDV serotype worldwide. This approach could yield a vaccine candidate that is both safe and immunogenic, with minimal risk of reversion to virulence. Here, we describe the design, generation, and characterization of a codon pair-deoptimized FMDV O strain, assessing its attenuation, genetic stability, and immunogenic potential in vitro and in vivo.

Our findings provide valuable insights into the feasibility of this strategy for the development of next-generation FMD vaccines.

## Results

### Codon pair deoptimization of the P1-coding region of serotype O FMDV results in viable FMDV with attenuation in cell culture

Previous studies have shown that FMDV serotype A and Asia 1 with codon pair deoptimized (CPD) P1 regions can be successfully rescued and are attenuated in cell culture^10^. In order to test whether this approach is tolerated by serotype O, we designed a viral genome in which codon pair usage in the P1-coding region was deoptimized by replacement with non-preferred codon pairs. The deoptimized sequence (Supplementary Table 1) contained 486 single nucleotide changes across the P1-coding region. Table 1 shows the distribution of CpG and UpA dinucleotides between the wild-type and the deoptimized sequence.

**Table 1:** Comparison of CpG and UpA dinucleotides between the wild-type and the deoptimized sequence. Frequency is expressed as the ratio of the observed frequency of each dinucleotide in the sequence to the frequency expected from the base composition of the sequence as calculated by SSE.

	Wild-type sequence	Deoptimized sequence
Total CpG	133	286
Frequency CpG	0.0604	0.1299
Total UpA	62	197
Frequency UpA	0.0282	0.0895

After transfection of purified plasmid DNA, viable OBUL-P1-CPD virus was successfully rescued. It was passaged on BHK-21 cells and further evaluated on LFBK-αvβ6 and BHK-21 cells in comparison to its parental strain O/FRA/2001-ORF(O/BUL/2011).

### OBUL-P1-CPD produces delayed and incomplete cytopathic effects in LFBK-αvβ6 cells

**Fig. 1:**
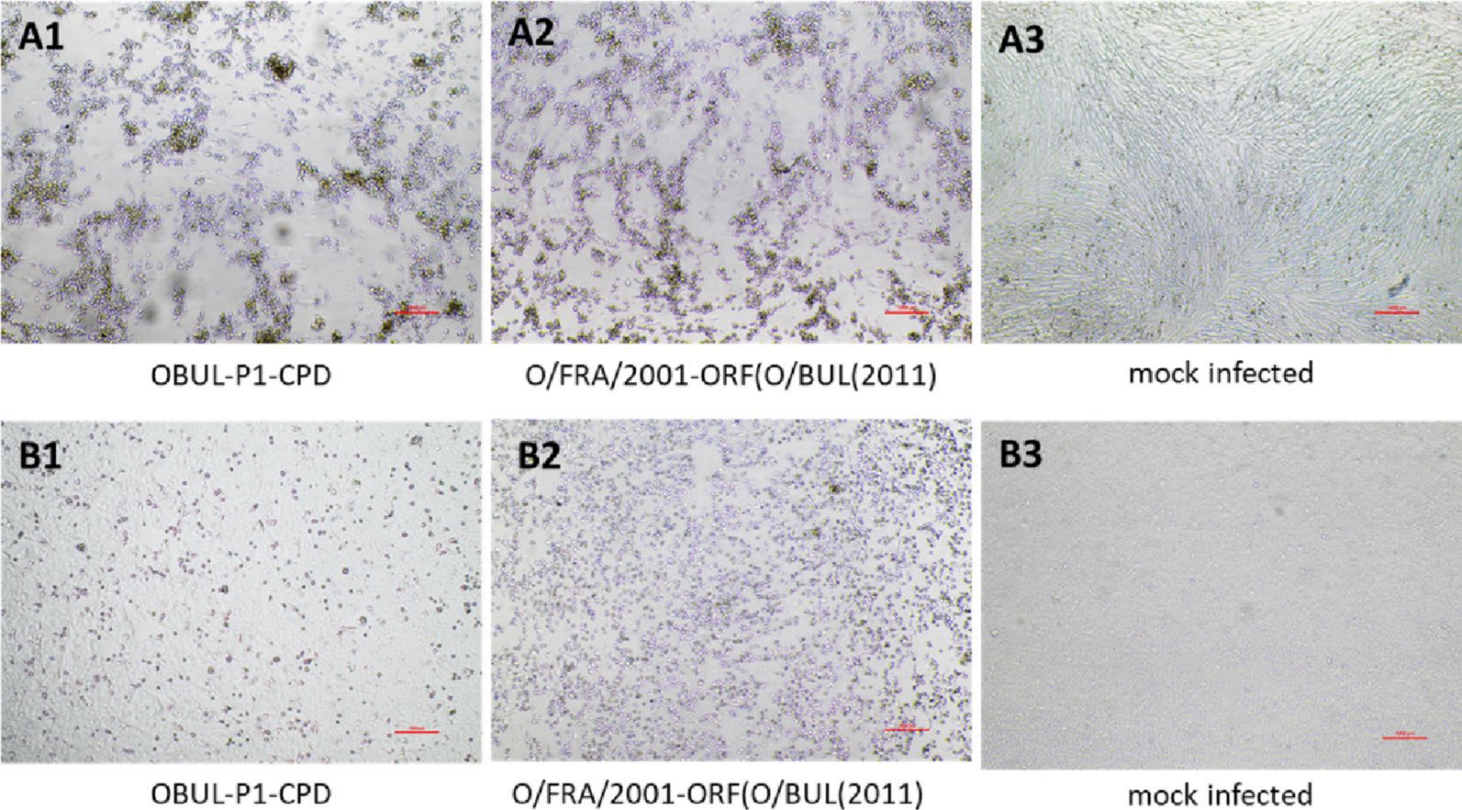
Cytopathic effects of OBUL-P1-CPD and its parental virus in BHK-21 and LFBK-αvβ6 cells at 20 h post-infection. BHK-21 and LFBK-αvβ6 cells were infected with either the CPD virus or its parental counterpart at an MOI of 0.1, with a mock-infected control included for reference. A: Both the CPD strain (**A1**) and its parental virus (**A2**) induce extensive cytopathic effects in BHK-21 cells, characterized by cell rounding and detachment. B: In LFBK-αvβ6 cells, the CPD strain (**B1**) shows markedly reduced CPE, indicative of attenuation in comparison to its parent virus (**B2**). Mock-infected BHK-21 (**A3**) and LFBK-αvβ6 (**B3**) cells remain intact, demonstrating normal cell morphology. Images were taken at 40x magnification to allow direct comparison.

In porcine LFBK-αvβ6 cells, OBUL-P1-CPD exhibited a delayed and incomplete onset of cytopathic effects (CPE) compared to O/FRA/2001-ORF(O/BUL/2011). While the parental strain induced rapid and complete CPE within 20 h post-infection, OBUL-P1-CPD showed a significantly slower progression of CPE, remaining incomplete even after 48 h. In contrast, no differences in CPE development were observed between OBUL-P1-CPD and the parental strain in BHK-21 cells, where both viruses induced rapid and complete CPE within 20 h post-infection (Fig. 1).

### Replication of OBUL-P1-CPD and O/FRA/2001-ORF(O/BUL/2011) in BHK-21 and LFBK-αvβ6 cells

**Fig. 2:**
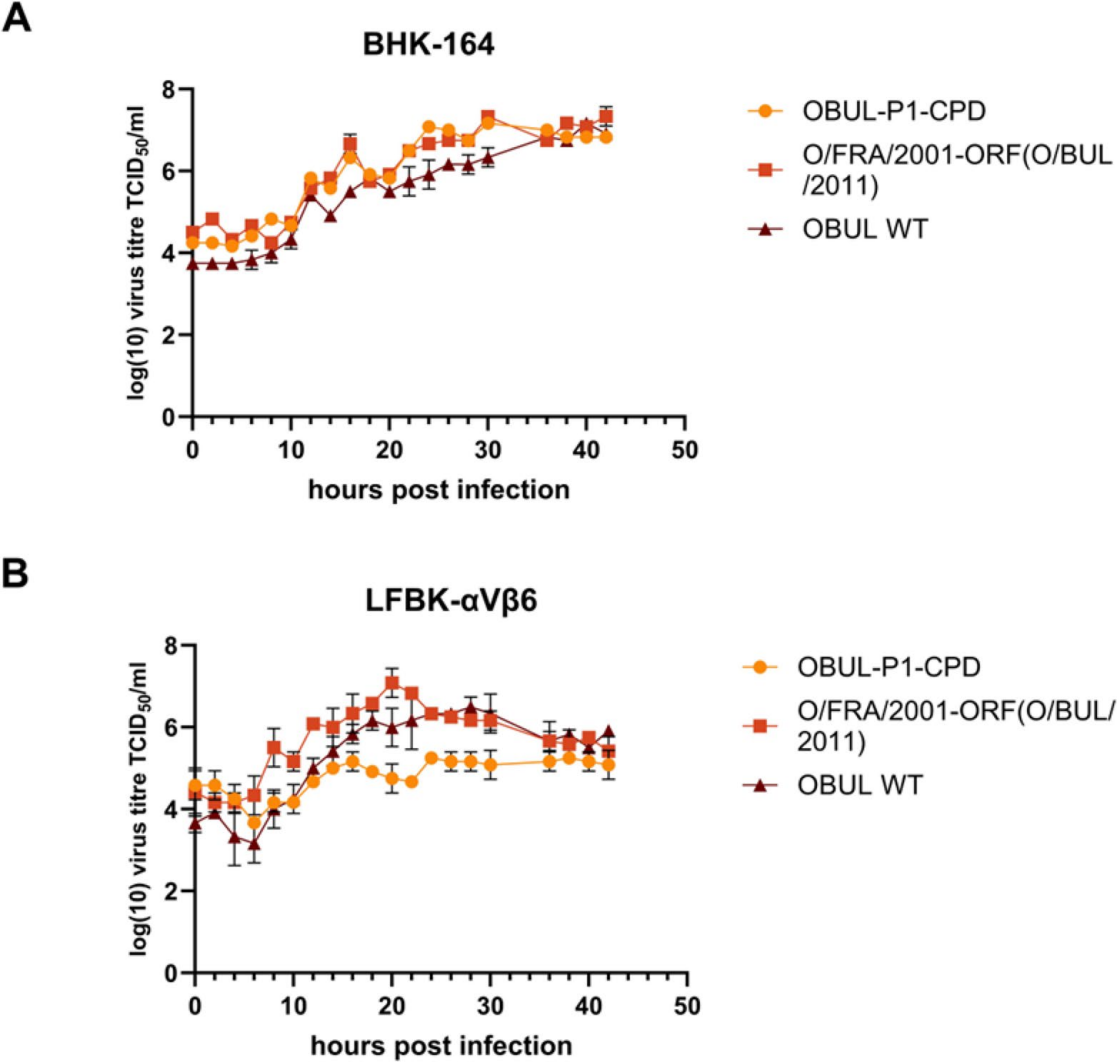
Growth kinetics of OBUL-P1-CPD, its parental virus O/FRA/2001-P1(O/BUL/2011) and the original isolate OBUL WT in BHK-21 (**A**) and LFBK-αvβ6 (**B**) cells. In BHK-21 cells, OBUL-P1-CPD, O/FRA/2001-ORF(O/BUL/2011) and OBUL WT exhibited similar growth profiles. In contrast, OBUL-P1-CPD showed significantly reduced replication in LFBK-αvβ6 cells. Mean ± SD of two biological replicates is shown.

Cultures of BHK-21 and LFBK-αvβ6 cells were inoculated with OBUL-P1-CPD, OBUL WT or O/FRA/2001-ORF(O/BUL/2011), harvested at different time points and the amount of virus present was determined by titration. Growth kinetics analyses revealed that OBUL-P1-CPD reached lower endpoint titers in porcine LFBK-αvβ6 cells compared to the parental strain. In LFBK-αvβ6 cells, maximum titers for O/FRA/2001-ORF(O/BUL/2011) were obtained after 20 h at 7.1 log10 TCID_50_, while maximum titers for OBUL-P1-CPD were reached after 24 h at 5.3 log10 TCID_50_ (Fig. 2). The reduction in viral titer indicates impaired replication efficiency in this cell line. However, in BHK-21 cells, the growth kinetics of OBUL-P1-CPD were similar to those of the parental virus, with no significant titer differences observed: both viruses reached a plateau at about 24 h at 7.1 (OBUL-P1-CPD) and 6.7 (O/FRA/2001-ORF(O/BUL/2011)) log10 TCID_50_ respectively.

### OBUL-P1-CPD shows reduced plaque size in LFBK-αvβ6 cells

**Fig. 3:**
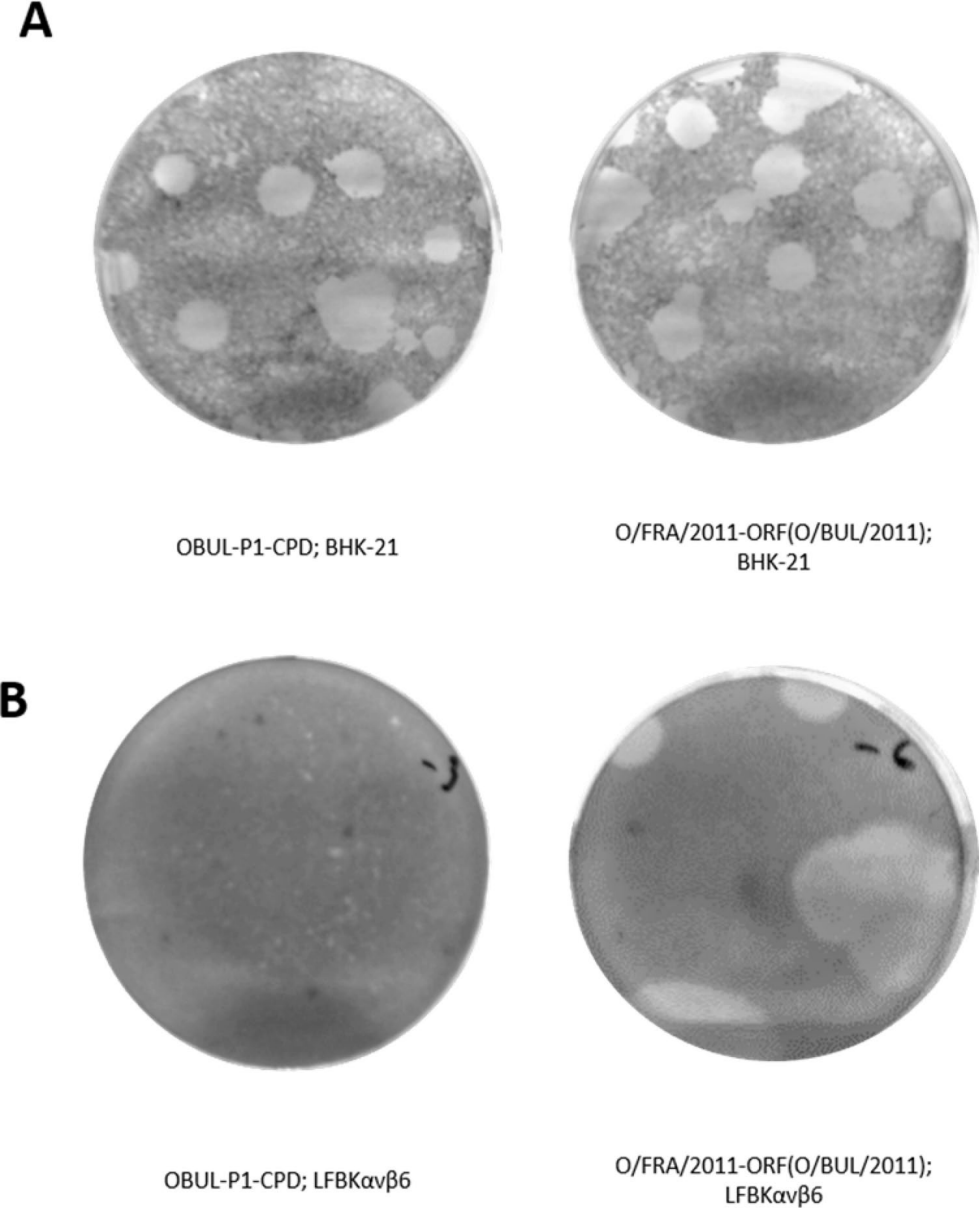
Plaque assays of OBUL-P1-CPD and its parent virus O/FRA/2001-ORF(O/BUL/2011) on BHK-21 and LFBK-αvβ6 cells. (**A**) In BHK-21 cells, plaque formation is similar for both OBUL-P1-CPD and O/FRA/2001-ORF(O/BUL/2011). (**B**) In porcine LFBK-αvβ6 cells, OBUL-P1-CPD forms significantly smaller plaques compared to the parental virus, indicating reduced viral replication and cytopathic effect. Brightness was adjusted uniformly across the entire image to improve visibility.

Plaque assays further demonstrated the attenuated phenotype of OBUL-P1-CPD in porcine LFBK-αvβ6 cells. The deoptimized virus formed approximately 100-fold smaller plaques compared to the parental O/FRA/2001-ORF(O/BUL/2011) strain (0.2 mm vs. 51.5 mm mean diameter), reflecting reduced viral replication and spread in this cell line (Fig. 3). Conversely, plaque sizes in BHK-21 cells were similar for both OBUL-P1-CPD and the parental virus, indicating that the attenuating effects of CPD were cell type-specific. There was no difference in plaque diameter between O/FRA/2001-ORF(O/BUL/2011) and OBUL-WT.

### Fluorescent Immunostaining

**Fig. 4:**
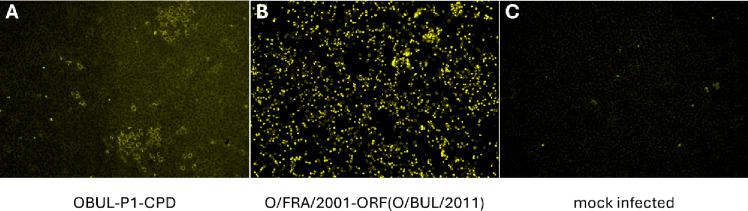
Fluorescent immunostaining of LFBK-αVβ6 cells with an anti-FMDV-3B antibody 21 h after infection with OBUL-P1-CPD or O/FRA/2001-ORF(O/BUL/2011). The cells were infected with either the CPD virus or its parent at an MOI of 0.01, with a mock-infected control included for reference. The CPD strain (**A**) shows much reduced cytopathic effect and fluorescent signal, indicative of lower FMDV replication, in comparison to its parent virus (**B**). Mock-infected LFBK-αvβ6 cells (**C**) remain intact and unstained.

To further evaluate the observed differences in growth kinetics of the CPD virus compared to its parent virus in the LFBK-αvβ6 cell line, we stained infected cultures with fluorescently labeled anti-FMDV antibodies. After 21 h, LFBK-αvβ6 cells infected with the CPD virus show only limited FMDV signal in individual cells and cell clusters, while the monolayer stays intact and attached. Wells incubated with the parent virus show strong fluorescence and the monolayer appears to be completely detached (Fig. 4).

### Evaluation of OBUL-P1-CPD in Pigs

To assess the attenuation and potential immunogenicity of OBUL-P1-CPD in vivo, an animal trial was conducted. Six crossbred pigs were intradermally injected with 0.1 mL of OBUL-P1-CPD into each primary digit of the right hind foot (0.2 mL in total), delivering a total dose of 1.3 × 10^5^ TCID_50_ per animal. None of the animals displayed any clinical signs of FMD within 21 days post-inoculation (dpi).

A second OBUL-P1-CPD inoculation was performed via the same route on day 21 with 0.2 mL of an increased dose of 4.7 × 10^6^ TCID_50_. Three days later, the first animals (#50 and #53) developed coronary band lesions characteristic of FMD as well as lameness in the inoculated limb. Additionally, one other animal (#48) showed a slight increase in body temperature (≥ 39.5 °C) on day three post inoculation. Between days four and six post inoculation, all but one of the remaining animals developed clinical signs of FMD, including vesicular lesions and lameness, consistent with an active FMDV infection. Pig #51 remained asymptomatic throughout the trial and was euthanized on day 27, as it was the last animal in the room. Table 2 shows the asynchronous progression of FMDV infection during the study.

**Table 2:**
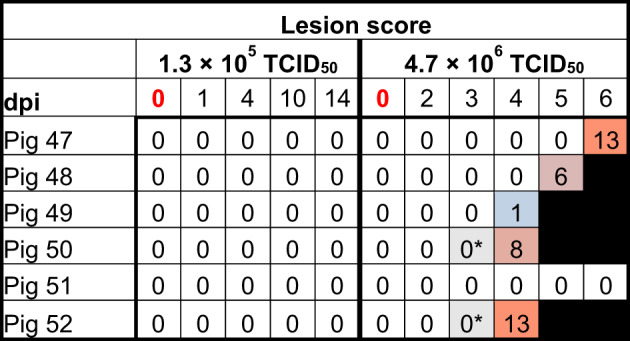
Lesion scores following two inoculations of OBUL-P1-CPD at different doses. The table presents the progression of lesions for each animal. The appearance of lesions on each digit, as well as on the snout, lips and oral cavity was recorded daily. The inoculated limb was disregarded, resulting in a maximum score of 15 points. 0* indicates an animal with lesions exclusively on the inoculated limb. Black cells signify that the animal had already been euthanized. Bold red text indicates inoculation days.

No FMDV RNA was detected in serum and oropharyngeal swab samples collected from the animals up to 21 days after low-dose inoculation with 1.3 × 10^5^ TCID_50_ of OBUL-P1-CPD. Following the high-dose inoculation with 4.7 × 10^6^ TCID_50_ of OBUL-P1-CPD, detectable viral RNA was present in serum and swab samples beginning at around day two (Table 3).

**Table 3:**
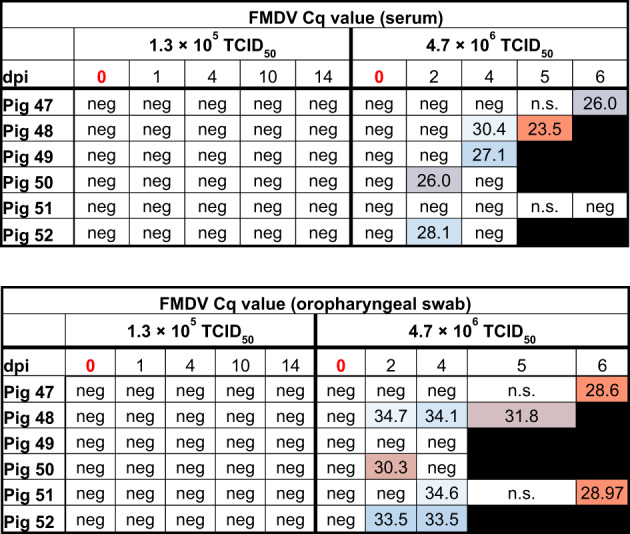
FMDV RT-qPCR results of serum and oropharyngeal swab samples following inoculations of OBUL-P1-CPD. Bold red text indicates inoculation days. Black bars signify that the animal had already been euthanized, “n.s.” indicates that no sample was collected from the animal on this day.

None of the animals were positive for FMDV-specific antibodies or neutralizing antibodies at the time of the second inoculation; however, pigs #50 and #52 exhibited low but detectable neutralizing antibody titers in the final sample taken 4 days later (see Table 4). The NSP and SP ELISA signal was similarly increasing, but had not reached the positive cut-off.

**Table 4:** Serological results are shown as S/N ratios for the NSP and type O SP antibody ELISAs (mean of two replicates) and as titers (final dilution) for the type O VNT. S/N values < 50 are positive. “Final” indicates the last sample taken from each animal at the time of euthanasia.

Pig	NSP ELISA 10 dpi	VNT 14 dpi	Type O ELISA 14 dpi	Type O ELISA 21 dpi	Type O ELISA final	VNT final
47	136	> 1:8	115	115	134	> 1:8
48	140	> 1:8	123	123	121	> 1:8
49	139	> 1:8	124	124	114	> 1:8
50	139	> 1:8	120	120	83	1:32
51	131	> 1:8	125	125	118	> 1:8
52	134	> 1:8	140	140	99	1:12

### Genetic stability of the deoptimized P1-coding region

To assess whether the observed pathogenicity of high-dose OBUL-P1-CPD was due to its inherent properties or caused by a genetic adaptation leading to increased virulence, viral RNA from lesion samples collected at 17 different locations and timepoints across five pigs was analyzed by Sanger sequencing. It was confirmed that the P1-coding region of OBUL-P1-CPD remained unchanged in all samples after in vivo replication. The nucleotide sequences of the recovered viruses matched the original deoptimized sequence with 100% identity, confirming the genetic stability of the CPD genome under the conditions of this study.

## Discussion

The development of new, safe and effective vaccines against FMD remains a global priority due to the high transmissibility and devastating impact of the virus. This proof-of-concept study explored the feasibility of codon pair deoptimizing of an FMDV serotype O strain to create live attenuated vaccines. By targeting the P1 region, which encodes the structural proteins^13^, CPD introduces silent mutations that reduce viral replication efficiency without altering the antigenic properties of the virus. Previous studies have successfully employed CPD to attenuate FMDV (and other viruses) using a “synthetic attenuated virus engineering (SAVE)” approach^14–16^. Essentially, this is an algorithm that recodes an amino acid sequence, resulting in underutilized codon pairs while preserving the codon bias and the folding free energy of the RNA. These studies demonstrated the viability of CPD as a strategy for live-attenuated vaccine development. However, concerning FMDV, previous efforts have been limited to serotypes A and Asia1, leaving serotype O, arguably the most widespread serotype of highest global significance, unexplored. Expanding CPD-based attenuation to serotype O could provide a critical advancement in FMDV vaccine development, particularly given the high prevalence and economic impact of this serotype. Our findings highlight both the promises and challenges associated with this approach.

The P1-coding region of FMDV O/BUL/2011 was selected for codon-pair deoptimization (CPD) rather than the non-structural protein (NSP)-coding regions for several reasons: First, P1 encodes the capsid proteins that are directly responsible for host cell receptor recognition, viral attachment, entry, and immune evasion^17,18^. Disruption of capsid protein translation efficiency or folding dynamics through CPD could theoretically attenuate viral infectivity while preserving the essential replication machinery of the NSPs. Second, NSP-coding regions encode critical enzymes essential for the viral life cycle inside the host cell^19^. Deoptimization of these regions could risk disrupting genome replication, resulting in a non-viable virus. Therefore, the P1-targeted deoptimization is the safest approach to achieve controlled attenuation that maintains sufficient replication for vaccine immunogenicity. Third, deoptimization targeting structural proteins has been shown to be effective in other RNA virus systems^14,20^. This approach allows evaluation of whether CPD-mediated attenuation of structural proteins alone is sufficient to generate protective immunity. Finally, the P1 region represents a substantial portion of the FMDV genome, providing ample opportunity for introducing silent mutations that collectively reduce viral fitness through cumulative codon-pair bias effects. The increased CpG and UpA frequencies in the deoptimized sequence we observed may drive attenuation by promoting host antiviral recognition and decreasing replicative fitness. Prior work suggests that these dinucleotide increases specifically, rather than codon-pair changes alone, lead to reduced viral fitness^21–23^.

Previous studies^24–27^ have explored possible conserved RNA secondary structures located within the open reading frame of FMDV (and other picornaviruses) and elucidated the importance of interactions between RNA secondary structures and capsid proteins during viral assembly. While some analyses have identified conserved putative packaging signals within the P1-coding region of FMDV^24^, others have found a host of conserved RNA structures within the regions coding for non-structural proteins^25^. To minimize the possible impact of CPD on viral assembly itself, we selected a CPD P1 sequence with RNA secondary structure elements closely resembling those of the wild-type virus.

The in vitro characterization of OBUL-P1-CPD demonstrated clear evidence of attenuation in porcine-origin LFBK-αvβ6 cells. Fluorescent immunostaining showed markedly reduced (NSP) protein production by OBUL-P1-CPD in LFBK-αvβ6 cells, correlating with delayed and incomplete CPE, smaller plaques, and lower endpoint titers compared to parental or wild-type viruses. Notably, this attenuated phenotype was not observed in BHK-21 cells, suggesting that the impact of CPD is likely cell type-dependent. LFBK-ανβ6 cells are derived from porcine kidney epithelium and were genetically engineered to stably express bovine ανβ6 integrin, the primary receptor for FMDV^28^. These cells show exceptionally high sensitivity for FMDV and often outcompete BHK-21 cells in clinical sample isolation and early onset of CPE^29^. Thus, LFBK-ανβ6 cells more closely mimic the natural host environment than BHK-21 cells, which are fibroblast-like cells of hamster origin with a deficiency in type I interferon responses^30^. In contrast, LFBK-ανβ6 cells retain the responsiveness to porcine type I interferons and their antiviral activity, showing at least some degree of innate immune competence^31^. The cell-type dependent attenuation raises important considerations for safety and efficacy. The lack of a detectable effect in BHK-21 cells suggests that the CPD-mediated attenuation may be dependent on a functional innate immune system. If the deoptimized virus replicates unhindered in the absence of an interferon response, this raises questions about the inherent stability of the attenuated phenotype and the reversion potential in cells with limited interferon competence. These differences in the innate immune response are a potential avenue for further exploration of the underlying mechanisms responsible for the cell-specific viral phenotypes observed in this study. Furthermore, this variability underscores the importance of selecting appropriate cell lines for the preclinical evaluation of vaccine candidates. While the vaccine prototype in this study was designed exclusively for use in pigs, the observed differences in cell lines of different origin raise questions about the safety in other natural FMDV hosts, such as cattle.

In vivo, the initial inoculation of pigs with 1.3 × 10^5^ TCID_50_ of OBUL-P1-CPD did not result in clinical disease nor detectable viral RNA or seroconversion, indicating that this dose was insufficient to establish infection with the vaccine candidate virus. Similar doses of other serotype O viruses lead to fulminant clinical FMD within 48 h^32^. However, in contrast to deoptimized viruses of other serotypes administered at similar doses^10^, no detectable seroconversion was observed in any of the animals in this study. Following a second inoculation with 4.7 × 10^6^ TCID_50_ of the type O CPD virus, five out of six animals exhibited clinical signs of FMDV infection, accompanied by detectable viral RNA and vesicular lesions, leaving the margin between innocuity and virulence surprisingly narrow. One constraint of our study design lies in the group housing of pigs, which allows infected animals to spread the inoculated virus to their pen mates. Given the staggered onset of clinical signs (and detectable FMDV RNA) within the group, it seems likely that not all animals had been primarily infected by the IDHB inoculation with 4.7 × 10^6^ TCID_50_ of OBUL-P1-CPD. Rather, the inoculation may have only been successful in approximately half of the animals and was then subsequently spread to the others. At the same time, this demonstrates the significant amount of shedding of the CPD virus (presumably originating mainly from the abundant vesicular lesions), in contrast to the observations of Medina et al.^10^. It is important to note in this context that RT-qPCR detects viral RNA rather than infectious virions. Infectivity assays such as plaque assays or virus titration could provide complementary information on the shedding and further strengthen the mechanistic interpretation of viral attenuation.

The absence of an antibody response to the initial inoculation aligns with the lack of detectable viral replication in the pigs at the tested dose of 1.3 × 10^5^ TCID_50_. This may reflect insufficient antigenic stimulation to elicit a measurable adaptive immune response. While neutralizing antibodies are considered the principal correlate of protection against FMDV, cell-mediated and innate immune responses also contribute to antiviral control^33,34^. This study did not characterize the induction of cell-mediated immunity or measure innate immune activation (e.g., interferon production) following inoculation. Thus, we cannot definitively exclude the possibility that partial immunity or heterologous protection mechanisms were induced but not detected by the serological methods we employed. However, the complete absence of a protective immunity upon high-dose secondary challenge suggests that any innate immune responses generated by the primary inoculation were insufficient to provide meaningful protection to begin with.

The second, higher inoculation dose (4.7 × 10^6^ TCID_50_), while eliciting clinical disease, also highlighted the potential of OBUL-P1-CPD to act as an immunogenic platform. Future studies could attempt to optimize dosing regimens and evaluate immune responses under subclinical conditions to determine whether the deoptimized virus can confer protective immunity without causing overt disease. Enhancing immunogenicity by the use of adjuvants may also prove to be a viable approach.

A critical aspect of any LAV is the genetic stability of the attenuated genome to minimize the risk of reversion to virulence^35–37^. The complete preservation of the deoptimized P1-coding sequence in OBUL-P1-CPD after in vivo replication provides first evidence for the stability of the introduced mutations. In theory, CPD-based approaches hold a significant advantage over other live-attenuated vaccine platforms, as the large number of silent mutations reduces the likelihood of reversion to virulence, as has been demonstrated for other RNA viruses^14,38,39^. Another important concern is the possibility of recombination events between an LAV and wild-type viruses. Recent studies of in vitro recombination with codon-deoptimized and wild-type FMDV concluded that recombination occurs despite the presence of deoptimized regions^40^. However, incorporation of deoptimized sequences always led to a reduction in fitness and stability of the recombinant genomes.

Ultimately, our proof-of-concept study is not suited to determine the genetic stability. Sustained viral replication during multiple animal passages could select for reversions or compensatory mutations that restore viral fitness and virulence. While no such mutations were seen in the P1-coding region in this study after one passage in vivo, further safety studies involving serial animal passages, combined with full-genome sequencing of passaged viruses, are essential to confirm genetic stability and definitively assess the reversion potential of the CPD viruses.

CPD-based LAVs offer a compelling alternative for FMD control, potentially combining rapid and long-lasting protection, mucosal immunity and DIVA compatibility. These traits are particularly valuable given the short incubation time and fulminant transmission capacity of FMD. In practice, the potential advantages differ between FMD-free and endemic regions: In FMD-free countries with intensive, high-value livestock production (e.g., in North America or Europe), a well-characterized safe LAV could provide quick protection of at-risk herds during emergencies or ring-vaccination scenarios, and likely at lower doses and cost than inactivated vaccines. This could reduce the need for large-scale culling and shorten trade embargoes, but the regulatory hurdles for FMD LAVs are high. In endemic countries, where routine vaccination must be able to cover large areas with often constrained infrastructure, thermostable CPD-LAVs would be of even more potential benefit: lower dose requirements, reduced reliance on costly antigen production in high containment, and the possibility of targeting hard-to-reach populations (e.g., smallholders or wildlife reservoirs) could make sustained control and eventual eradication of FMD more realistic.

Our findings demonstrate the feasibility of using CPD to attenuate FMDV serotype O and provide a foundation for further development of live attenuated vaccines and safe vaccine backbones. However, several challenges remain: First, the cell-specific attenuation observed in vitro highlights the need for additional studies to understand how CPD affects viral fitness in different host cell environments. Second, the dose-dependent pathogenicity observed in vivo underscores the importance of refining CPD designs to properly attenuate the virus while preserving its immunogenicity.

## Materials and methods

### Cell lines

Baby hamster kidney (BHK-21) cells (CCLV-RIE 0164, Collection of Cell Lines in Veterinary Medicine, FLI, Greifswald-Insel Riems, Germany) were used for both viral propagation and transfection experiments. A T7 polymerase-expressing clone of BHK-21 cells, known as BSR-T7 cells (CCLV-RIE 582)^41^, was used for transfection and virus rescue.

BHK-21 cells were maintained in a growth medium composed of Minimum Essential Medium (MEM) supplemented with Hank’s salts and non-essential amino acids. BSR-T7 cells were cultured in a nutrient-rich medium containing tryptose phosphate, casein peptone, meat peptone, and yeast extract. Both media were supplemented with 10% fetal bovine serum (Cytiva, Marlborough, USA). The cells were incubated at 37 °C in a 5% CO₂ atmosphere.

Porcine LFBK-ανβ6 (CCLV-RIE 1419) were used for viral propagation and maintained in a growth medium composed of Iscove’s Modified Dulbecco’s Medium supplemented with Ham’s F12-Medium in a 1:1 ratio.

### Viruses and plasmids

The FMD field strain used in this study was initially isolated from a wild boar in Bulgaria (strain designation O/BUL/HS018-1/2011, accession number PQ619438, hereafter referred to as the wildtype “OBUL WT”). It was provided by the Bulgarian National Veterinary Service^42^. The nucleotide sequence of its polyprotein open reading frame (ORF) was determined using material from the second passage on LFBK-ανβ6 cells.

### Generation of codon-pair-deoptimized (CPD) FMD virus

A codon-pair–deoptimized FMDV was generated using a restriction-free cloning approach based on a recoded P1 region of FMDV O/BUL/2011. Full details of the recoding strategy, cloning procedure, and virus rescue are provided in the Supplementary Methods S1. Briefly, codon pair deoptimization was performed using Simple Sequence Editor (SSE, V1.4)^43^ with a porcine codon pair usage table, aiming to minimize the codon pair score (i.e., incorporating the most underrepresented codon pairs). The deoptimized P1 region was subsequently cloned into the previously constructed infectious clone pT7S3_O/FRA/2001-ORF(O/BUL/2011) and the deoptimized virus OBUL-P1-CPD was rescued as described.

### Viral growth kinetics

The viral growth kinetics of the chimeric virus O/FRA/2001-ORF(O/BUL/2011), its deoptimized derivative OBUL-P1-CPD and the original OBUL WT virus isolate were compared at a multiplicity of infection (MOI) of 0.1. This comparison was conducted on two cell lines: BHK-21 (hamster) and LFBK-αvβ6 (pig). Samples were collected every two hours for 0–30 h post-infection (hpi) and at 36, 38, 40 and 42 hpi and subsequently titrated on BHK-21 cells. Mock-infected controls (one well per plate) received virus-free medium and were processed in parallel with infected samples. Mean values and standard deviations were calculated from two independent experiments using GraphPad Prism version 10.2.1 (GraphPad Software, San Diego, USA).

### Plaque assay

Confluent cultured monolayers of BHK-21 cells and LFBK-αvβ6 cells were prepared in 6-well plates. One hundred µL of a ten-fold dilution series of the viruses were inoculated per well and incubated at 37 °C with 5% CO_2_ for 1 h. The supernatant was discarded and the cells were overlaid with 2 mL of 1% methyl cellulose (Carl Roth, Karlsruhe, Germany) in MEM and incubated for approximately 60 h. The overlay was discarded, wells were washed two times with phosphate-buffered saline and stained with 1% crystal violet in 10% formaldehyde for 1 h, after which the staining solution was discarded and the wells were rinsed with water. After drying, plates were imaged with the GEL iX Imager (INTAS, Göttingen, Germany) and plaque diameters were measured using ImageJ (version 1.54 g; National Institutes of Health, Bethesda, USA).

### Fluorescent immunostaining of FMDV-infected cell cultures

Confluent monolayers of LFBK-ανβ6 cells in 24-well plates were infected at an MOI of 0.01 with OBUL-P1-CPD or O/FRA/2001-ORF(O/BUL/2011) and incubated at 37 °C with 5% CO_2_. After 21 h, plates were processed by first removing the supernatant and washing the wells once with PBS. Cells were then fixed with 4% formaldehyde in PBS for 20 min, followed by one wash with PBS. Permeabilization was performed with PBS containing 0.1% Triton X-100 (PBS/T) for 5 min. Subsequently, cells were incubated with anti-FMDV monoclonal antibody 9F2 targeting 3B (kindly provided by Sven Reiche, Friedrich-Loeffler-Institut), at 1:200 dilution in PBS/T containing 0.01% Tween 20) for 20 min. After two washes with PBS/T, cells were incubated with Alexa Fluor 546-conjugated anti-mouse IgG (Thermo Fisher Scientific, Dreieich, Germany) (1:500 dilution in PBS/T) for 20 min. Cells were then washed three times with PBS/T. Finally, PBS was added to each well prior to image acquisition with a fluorescence microscope. To increase the contrast of the immunofluorescence images, the black and white points were adjusted using Corel PHOTO-PAINT. The same setting was used for all images.

### Animal housing and care

All animal procedures were approved by the State Office for Agriculture, Food and Fisheries of Mecklenburg-Vorpommern (LALLF M-V file no. 7221.3-1-039/23). Six clinically healthy, crossbred pigs (Sus scrofa domesticus), aged 8 weeks and weighing approximately 20–25 kg, were obtained from a commercial swine breeding facility in Germany. The pigs were housed in the animal facilities at the Friedrich-Loeffler-Institut under veterinary biosafety level 4 conditions. The facility is equipped with group pens, each housing six animals, allowing for social interaction between the pigs. Temperature (20–22 °C) and relative humidity (50–60%) are controlled. Pigs were acclimated for one week prior to the start of the experiment, with ad libitum access to water and were fed once a day with a commercial swine diet.

### Experimental design

The OBUL-P1-CPD viral inoculum was prepared by diluting the virus in culture media to a final concentration of 6.5 × 10^5^ TCID_50_/mL (corresponding to a dose of 1.3 × 10^5^ TCID_50_/pig) for the first inoculation. For the second inoculation, undiluted viral OBUL-P1-CPD cell culture supernatant was used, resulting in a dose of 4.7 × 10^6^ TCID_50_/pig. All doses were confirmed by back titration of leftover inoculum after inoculation. Prior to inoculation, pigs were sedated by intramuscular (i.m.) injection with medetomidine (Domitor, Vetoquinol, Ismaning, Germany), midazolam (Midazolam-ratiopharm, Ratiopharm, Ulm, Germany) and butorphanol (Torbugesic Vet, Zoetis Deutschland, Berlin, Germany) (0.06, 0.3 and 0.3 mg/kg, respectively). All pigs were inoculated by injecting approximately 0.1 mL of viral suspension into the plantar skin of each primary digit of the right hind foot as previously described^44^, for a total volume of 0.2 mL. After successful inoculation, the anesthesia was reversed by i.m. injection of 0.3 mg/kg atipamezole (Antisedan, Vetoquinol) per pig.

### Monitoring and sampling

**Fig. 5:**
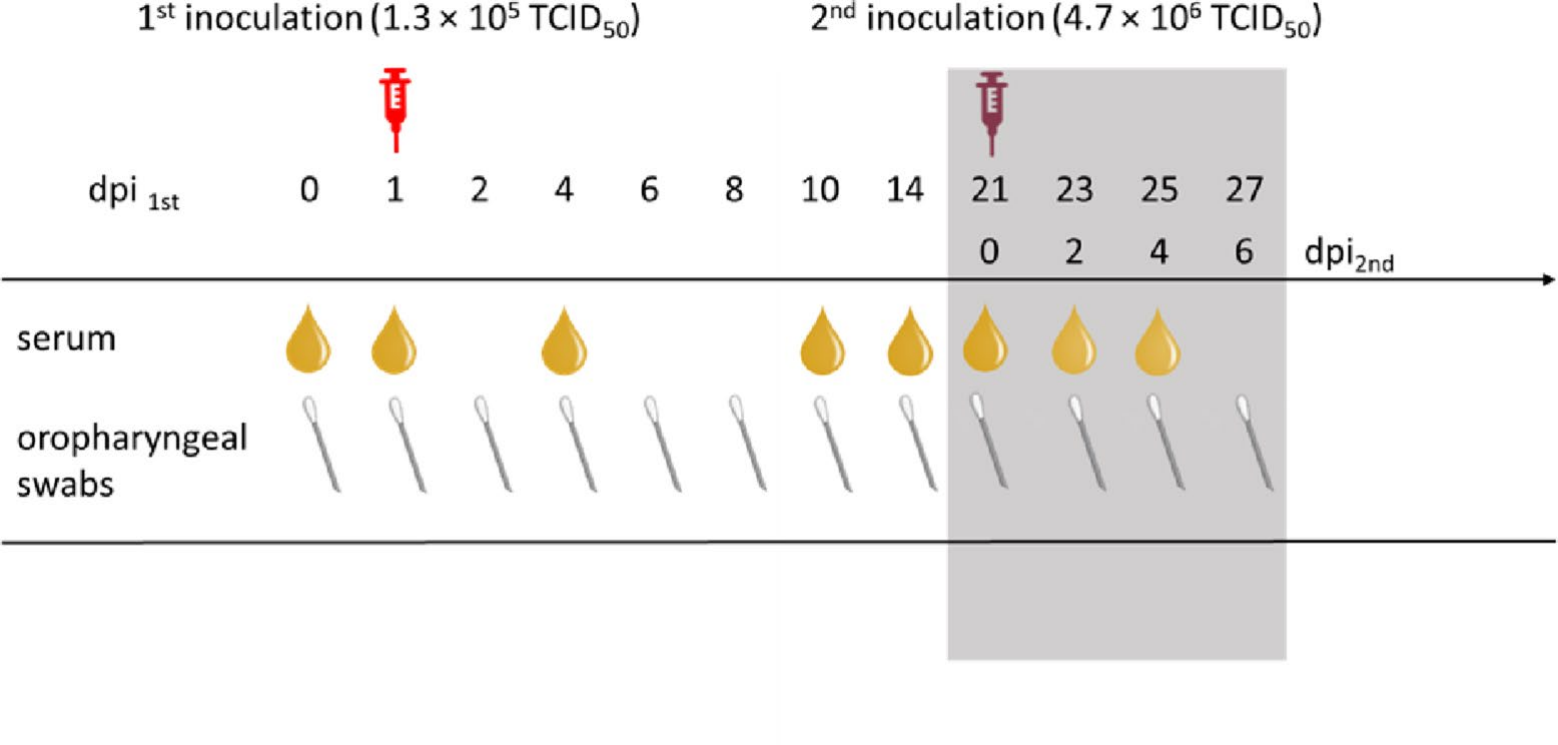
Overview of sampling scheme.

Clinical signs, rectal body temperature and feed intake were monitored daily throughout the experiment. Blood was collected via jugular venipuncture and oropharyngeal swab samples were taken with oversized rayon swabs (VWR International, Darmstadt, Germany) as shown in Fig. 5. Animals were examined daily for the appearance of lesions characteristic of FMD and scored; one point for each affected digit, as well as one point each for lesions on the snout, lips or in the oral cavity. The inoculated limb was disregarded, resulting in a maximum score of 15 points.

### Euthanasia and tissue collection

Pigs were euthanized once signs of generalized FMDV infection became apparent. For euthanasia, the animals were deeply anesthetized by intramuscular injection of tiletamine and zolazepam (Zoletil, Virbac, Bad Oldesloe, Germany), xylazine (Xylavet, CP‑Pharma, Burgdorf, Germany) and ketamine (Ketamin 10%, Serumwerk Bernburg, Bernburg, Germany) (3 mg/kg, 3 mg/kg, 1 mg/kg and 8 mg/kg, respectively), followed by intracardial injection of 0.12 mL per kg of T61 (Intervet Deutschland, Unterschleißheim, Germany; containing 200 mg/mL embutramide, 50 mg/mL mebezonium iodide and 5 mg/mL tetracaine hydrochloride). For animal welfare reasons, the last remaining pig was euthanized without showing any signs of FMDV infection. Additional serum and oropharyngeal swab samples were taken at the time of death, as well as swabs and lesion material from each affected limb.

### RNA extraction from liquid samples and tissues

Tissue samples taken prior to euthanasia were macerated in 2 mL Safe-Lock tubes (Eppendorf, Hamburg, Germany) containing 1 mL of MEM using a 5-mm steel ball in a TissueLyser II (Qiagen, Hilden, Germany) for 3 min at 30 Hz. After maceration, the samples were centrifuged to obtain clear supernatant. RNA was extracted from 100 µL of serum, oropharyngeal swab samples and tissue supernatants using the NucleoMag Vet kit (Macherey-Nagel, Düren, Germany) with a King Fisher Flex magnetic particle processor (Thermo Fisher Scientific, Dreieich, Germany). Ten µL of IC2 RNA were added during the extraction as an internal control^45^.

### Virus quantification

Quantification of viral load in serum and oropharyngeal swab samples was conducted by quantitative reverse transcription PCR (qRT-PCR) using specific primers and probes targeting the FMDV 3D-coding region, as previously described^46^.

### Serology

Antibodies against non-structural proteins (NSP) of FMDV were detected using the ID Screen FMD NSP Competition ELISA (Innovative Diagnostics, Grabels, France) according to the manufacturer’s instructions. Neutralizing antibodies were detected by a standard virus neutralization assay with FMDV O_1_ Manisa, as described in the Manual of Diagnostic Tests and Vaccines for Terrestrial Animals of the World Organisation for Animal Health.

### Sanger sequencing

RNA extracted from tissue samples was reverse transcribed and amplified with specific primers targeting the P1 region (listed in Supplementary Table 1) using the SuperScript III One-Step RT-PCR System with Platinum Taq DNA Polymerase (Thermo Fisher Scientific) according to the manufacturer’s instructions. PCR products were sent to Eurofins Genomics (Ebersberg, Germany) for Sanger sequencing using custom primers (listed in Supplementary Table 1).

## Supplementary Information

Supplementary Material 1

## Data Availability

The datasets generated and/or analysed during the current study are available in the GenBank repository at https://www.ncbi.nlm.nih.gov/nuccore/PQ619438 (FMDV sequence O/BUL/HS018-1/2011) and https://www.ncbi.nlm.nih.gov/nuccore/PX672754.1 (OBUL-P1-CPD).
