# Supplementary material for: Attenuation without protective immunogenicity: effects of codon-pair deoptimization on foot-and-mouth disease virus serotype O in swine
Source: Sci Rep. 2026 Jul 16;16:22288. doi: 10.1038/s41598-026-61879-8 (PMC13376744; doi:10.1038/s41598-026-61879-8)
Supplement: Supplementary file 1 — Supplementary Material 1 [file 41598_2026_61879_MOESM1_ESM.pdf]

Supplementary Methods S1

Generation of codon-pair-deoptimized (CPD) FMD virus

For the generation of a codon deoptimized FMD virus a restriction free cloning approach was used. Codon pair deoptimization was performed using Simple Sequence Editor (SSE, V1.4 [1]). Since SSE defaults to using the human codon pair usage table, it was instead configured to use that of the pig (Sscrofa11.1, NCBI RefSeq #GCF_000003025.6). The P1 region of FMDV strain O/BUL/2010 was recoded using SSE's "Mutate Sequences" function, aiming to minimize the codon pair score (i.e., incorporating the most underrepresented codon pairs). Since it did not conserve codon bias (or the codon adaptation index), codon usage also changed by introducing underrepresented codons.

The selected sequence was constructed with 3' and 5' overlapping ends corresponding to the sequence of O/BUL/HS018-1/2011 from an external vendor (Thermo Fisher Scientific GmbH, Dreieich, Germany) and subsequently cloned into the previously constructed infectious clone pT7S3_O/FRA/2001-ORF(O/BUL/2011) containing the open reading frame (ORF) of FMDV/O/BUL/2011 with the 3' and 5' untranslated regions of FMDV/O/FRA/2001. The pT7S3_O/FRA/2001-ORF(O/BUL/2011) plasmid was constructed via restriction free cloning [2] of FMDV/O/BUL/2011 into the previously constructed OBUL-GFP-Replicon-plasmid: A double-stranded DNA fragment corresponding to the ORF of FMDV/OBUL/2011, in which the P1-region had been replaced by a gene encoding green fluorescent protein (GFP) has been obtained from GeneScript (GenScript Biotech Corporation (Nanjing, China) and cloned into the previously constructed plasmid pT7S3-OFRA [3] as described below.

This OBUL-P1-CPD-megaprimer was combined with the pT7S3_O/FRA/2001-ORF(O/BUL/2011) plasmid in a PCR reaction (Phusion Hot Start Flex DNA Polymerase (New England Biolabs (NEB), Ipswich, USA) using the megaprimer in a 1:20 vector to insert molar ratio in a 20 µl reaction. The PCR product was incubated with DpnI (NEB) for 2 h at 37 °C and then transformed into 10-beta competent E. coli (NEB). After incubation for approximately 14 hours at 37 ° C, 24 colonies were transferred to 3 ml vials containing Luria Broth (25 g/l, Invitrogen) and 1 % ampicillin (100 mg/ml, Sigma-Aldrich, Merck KGaA, Darmstadt, Germany) (LB-Amp) and incubated for approximately 14 hours at 37 ° C. Plasmid DNA from 2 ml of solution was extracted and purified via a commercially available spin column system (Monarch Plasmid Miniprep Kit, NEB). Purified DNA was digested with KpnI and PvuII in rCutSmart Buffer (all NEB) according to the manufacturer's instructions. Digested samples were screened via electrophoresis on a gel containing 1 % agarose (Biozym, Hessisch Oldendorf, Germany) in TAE-Buffer (40 mM Tris-acetate, 1 mM EDTA, pH 8.0). Colonies containing the expected band distribution patterns were scaled up in 200 ml LB-Amp, plasmid DNA was extracted and purified as previously described and correct plasmid sequence was confirmed via Sanger sequencing.

Transfection of BSR-T7 cells in a 24-well plate was performed using purified plasmid DNA. Each well was transfected with 500 ng of DNA and 1.5 µl of Lipofectamine 3000 (Thermo Fisher Scientific GmbH, Dreieich, Germany). The virus was subsequently passaged three times on BHK-21 cells, with the cells undergoing a freeze-thaw cycle after cytopathic effects (CPE) were visible. For the passages 250 µl of the supernatant was used to infect fresh cells. CPE was observed from the first passage onwards. All virus stocks were sequenced using

Sanger sequencing and were stored at -80 °C. Virus titers were determined on BHK-21 cells by TCID50 assay (Spearman-Kärber method).

References

1. Simmonds, P. SSE: a nucleotide and amino acid sequence analysis platform. *BMC research notes* **5,** 50 (2012).

2. Bond, S. R. & Naus, C. C. RF-Cloning.org: an online tool for the design of restriction-free cloning projects. *Nucleic acids research* **40,** W209-13 (2012).

3. Litz, B. *et al*. Leaderless foot-and-mouth disease virus serotype O did not cause clinical disease and failed to establish a persistent infection in cattle. *Emerging microbes & infections* **13,** 2348526 (2024).

Supplementary Table S2: P1-CPD and primers covering the ORF of OBUL-P1-CPD

Name	Sequence
P1-CPD	5'-<br>GGCGCCGGACAATCTAGCCCCGCTACCGGTTCGCAAAACCAGTCCGGTAATACCGGTTCGATCATTAACAATTACTATATGCAACAGTATCAGAATTCGATGGATACGCAATTGGGCGATAACGCTATTAGCGGCGGATCGAACGAAGGTAGTACCGATACTACGAGTACGCATACGACGAATACGCAAAATAACGATTGGTTTTCGAAACTCGCATCTAGCGCTTTTAGCGG<br>ACTGTTCGGCGCACTGTTAGCCGATAAGAAAACCGAAGAGACTACACTCCTCGAGGATCGCATACTGACTACGCGTAACGGACATACGACGAGTACGACGCAATCTAGCGTAGGCGTAACGTACGGATACGCTACGGCCGAAGATTTCGTTAGCGGACCGAATACGTCCGGTCTCGAAACTCGCGTAGTGCAAGCCGAACGGTTTTTTAAGACGCATCTGTTCGATTGGGTT<br>ACGTCCGATTCGTTCGGTAGGTGTCATCTGCTCGAACTGCCAACCGATCATAAGGGCGTATACGGATCGCTTACCGATAGCTACGCTTATATGCGTAACGGTTGGGATGTCGAAGTTACGGCCGTAGGTAATCAGTTTAACGGCGGATGTCTGTTAGTCGCTATGGTACCCGAACTGTGTTCGATTTCGAAACGCGAACTGTATCAGCTTACGCTATTTCCGCATCAATTTATCA<br>ATCCGCGTACGAATATGACCGCACACATTACCGTACCGTTTATCGGCGTAAATCGGTACGATCAGTATAAGGTACACAAACCGTGGACACTCGTCGTTATGGTCGTCGCACCGCTTACGGTTAATACCGAAGGCGCACCGCAAATTAAGGTATACGCTAATATCGCACCTACGAACGTACACGTCGCAGGCGAATTTCCGTCGAAAGAGGGGATACTGCCCGTCGCATGTAGC<br>GACGGATACGGCGGACTCGTTACGACCGATCCGAAAACGGCCGATCCCGTATACGGTAAGGTATTCAATCCCCCCCGTACGATGCTCCCCGGTAGGTTTACGAATTTCCTCGACGTAGCCGAAGCATGCCCTACGTTTCTGCATTTCGAGGGAGACGTACCGTACGTTACGACTAAGACCGATAGCGATCGCGTACTCGCGCAATTCGATCTGTCGCTTGCCGCAAAGCATATG<br>TCGAATACGTTCCTTGCCGGACTCGCGCAATACTATACGCAATATAGCGGTACGATCAATCTGCATTTTATGTTTACCGGACCGACCGATGCGAAAGCTAGGTATATGATCGCATACGCTCCCCCCGGTATGGAACCGCCTAAGACACCCGAAGCGGCCGCACATTGCATACACGCCGAATGGGATACCGGACTGAATTCGAAATTTACGTTCTCAATCCCATACCTTAGCGCCG<br>CCGATTACGCATACACCGCTAGCGACGCAGCCGAAACTACGAACGTACAGGGGTGGGTTTGTCTGTTTCAGATTACGCACGGTAAGGCCGACGGAGACGCACTCGTAGCGTTAGCTAGCGCCGGTAAGGATTTCGAATTACGCTTACCCGTAGACGCACGTACGCAAACTACGAGTACGGGCGAATCCGCCGATCCCGTTACCGCAACCGTCGAGAATTACGGAGGCGAAA<br>CGCAAGTGCAACGTAGGCAACATACGGACGTATCGTTCATACTCGATAGGTTCGTTAAGGTTACGCCTAAGGATCAGATTAACGTACTCGATCTGATGCAAACCCCCGCTCATACACTCGTAGGCGCACTGTTACGCACCGCTACGTATTACTTCGCCGATCTCGAAGTCGCCGTTAAGCACGAAGGTAACCTTACGTGGGTACCGAATGGCGCACCCGAAACCGCACTCGATA<br>ATACGACGAATCCGACCGCATACCATAAGGCACCGCTTACGCGGTTAGCGTTACCGTACACCGCACCGCATCGCGTACTCGCAACCGTATACAACGGCGATTGTAAGTATAGCGAATCGCGTACTACGAACGTTAGGGGCGATCTGCAGGTACTCGCGCAAAAGGCCGCACGTACGTTACCGACTAGCTTTAATTACGGCGCAATCAAAGCGACTCGCGTTAACGAACTGTTA<br>TACCGTATGAAACGCGCCGAAACGTATTGTCCGCGACCGTTACTCGCAATACACCCTAACGAAGCTAGGCATAAGCAGAAAATCGTCGCACCCGTTAAGCAA-3'
3'UTR-OFRA-R1	5'-GCCTCAGCGTCTTTCTGCC-3'
FMD-1174-F	5'-TGGWGACAGGCTAAGGATGCC-3'
FMD-1889-F	5'-TTCTGGTRTTTGTYCCGTACGA-3'
FMD-4249-F	5'-GCAGGRGACGTBGAGTCCAA-3'
FMD-4303-R	5'-TGACGTCRGAGAAGAAGAARGG-3'
FMD-5143-F	5'-AGAACYGAYTCAGTTTGGTACTG-3'
FMD-5191-R	5'-GACCCYGACCACTTYGACG-3'
FMD-6143-F	5'-AACCRCAAGCTGAAGGACCCT-3'
FMD-6347-R	5'-CCTGTCGCTTTGAAAGTGAAAGC-3'
FMD-6567-F	5'-TGACTWCAGAGTGTTTGAGTTTGA-3'
FMD-7491-F	5'-CCARACCTTCCTGAAGGACGA-3'
FMD-7608-R	5-'TACACYAGRATGATGATTGGCAGA-'3
FMD-809-F	5'-ATGGGACGTCTGCGCACG-3'
FMD-8285-R	5'-AACTTCTCCTGKATGGTCCCA-3'
OBUL-P1-CPD-1570-F	5'-GTGGTACGCGATCGACGAC-3'
OBUL-P1-CPD-2032-R	5'-TGCGTCGTACTCGTCGTATG-3'
OBUL-P1-CPD-2412-F	5'-ATGACCGCACACATTACCGT-3'
OBUL-P1-CPD-2619-R	5'-CGGGCAGTATCCCCTCTTTC-3'
OBUL-P1-CPD-3025-F	5'-CACATTGCATACACGCCGAA-3'
OBUL-P1-CPD-3234-R	5'-CGAAATCCTTACCGGCGCTA-3'
OBUL-P1-CPD-3609-F	5'-TTAGCGTTACCGTACACCGC-3'
OBUL-P1-CPD-4083-R	5'-CAGTGGCCAGTTCCTCAAGT-3'
